# Trichomycoses

**DOI:** 10.4103/0974-7753.58552

**Published:** 2009

**Authors:** G Sentamilselvi, C Janaki, Sundaram Murugusundram

**Affiliations:** Department of Dermatology, Madras Medical College (Retd.), Chennai - 600 003, India; 1 Department of Consultant Dermatologist, Chennai - 600 010, India

**Keywords:** Malassezia folliculitis, piedra, tinea capitis, trichomycoses

## Abstract

Hair infection by fungal agents, also called trichomycoses, is one of the common concerns in human beings. The common agents causing hair infections are dermatophytes, Malassezia species and those causing piedra. The former two can give rise to considerable discomfort and also cause immune-mediated reactions in the form of kerion and dermatophytids. The etiopathogenesis of trichomycoses, along with its clinical aspects and the management, are briefed here.

## INTRODUCTION

Trichomycoses by definition are diseases of the hair caused by fungi. This field of trichology is gaining importance because of the following facts. Human trichomycoses cause great concern due to the cosmetic problem of loss of hair. The antigens of these fungi can induce severe inflammation, which may disable the persons. Vellus hair infection in sites other than the scalp may also give rise to recurrent episodes of infection and may maintain chronicity. There may be infection of the siblings and schoolmates in residential schools, especially with tinea capitis, and endemicity of the infection in Chennai has also been reported.[1‐3] Finally, hair infection has been suggested to be the portal of entry for deep mycosis due to dermatophytes.[4,5] Trichomycosis of the animals, although not described here, is also important because of the spread of infection to human and other animals and because the infected hide could be one of the causes for considerable economic loss in leather industries.

## PATHOMECHANISM OF TRICHOMYCOSIS BY VARIOUS FUNGI

The pathogenic fungi affecting the follicular units are mostly dermatophytes, agents causing piedra and Malassezia spp [Tables 1–3]. Dermatophytes and piedra directly invade the hair.

**Table 1:** Dermatophytes frequently causing trichomycosis

*Trichophyton violaceum*
*Trichophyton tonsurans*
*Trichophyton simii*
*Trichophyton rubrum*
*Trichophyton mentagrophytes*
*Trichophyton schoenleinii*
*Trichophyton yaoundei*
*Microsporum audouinii*
*Microsporum canis*
*Microsporum gypseum*
*Microsporum ferrugineum*

The dermatophytes causing trichomycosis may be anthropophilic (human), zoophilic (animal) or geophilic (soil). The ability of the dermatophytes to grow on the hair can easily be demonstrated by the hair bait technique,[6] which involves placing the sterile (acetone treated and dried) hair in a Petriplate containing soil. The dermatophytes present in the soil start invading the hair and produce colonies using hair as the substrate, showing their affinity for the hair. Some of the dermatophyte species can also produce perforating organs in the hair shaft.

The dermatophytes causing trichomycosis are listed in Table 1. *Trichophyton violaceum* has been observed as the most common agent causing tinea capitis in India.[7] Infection of the hair can occur on any hair-bearing area on the body surface.

**Figure 1:**
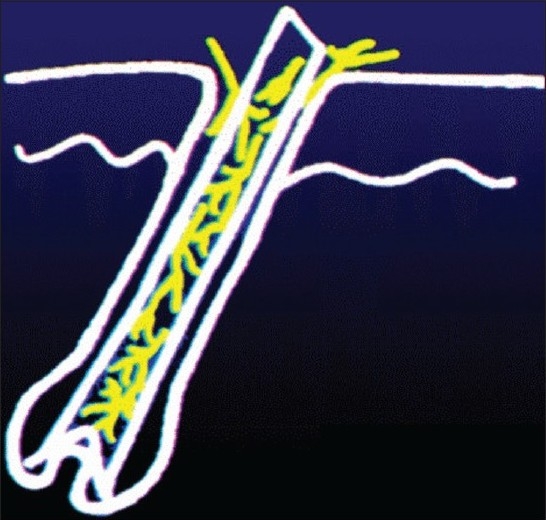
Intrapilary hyphae invading the hair cortex-schematic depiction

**Figure 2:**
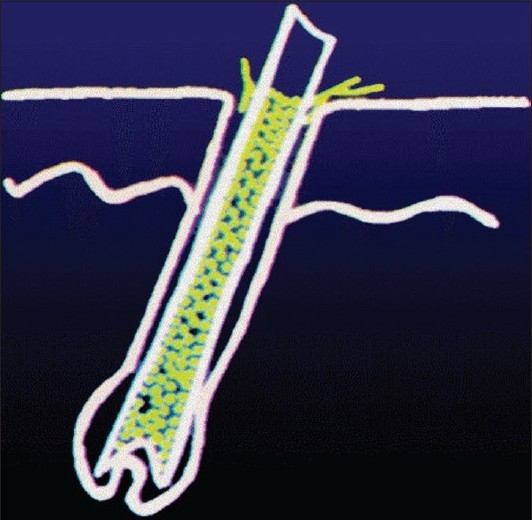
Endothrix spores-schematic depiction

**Figure 3:**
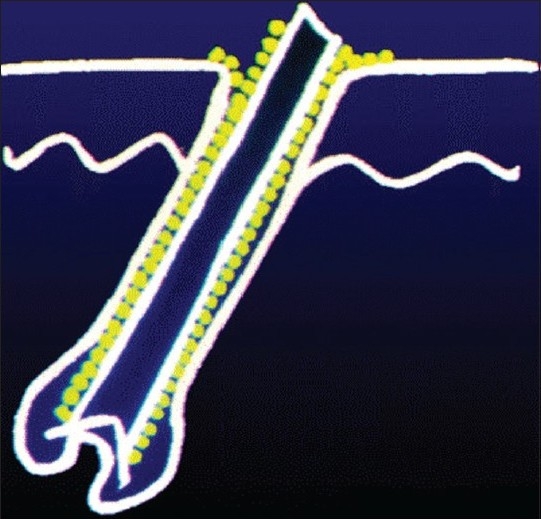
Ectothrix spores-schematic depiction

In human infection, the spores that get deposited on the stratum corneum invade the hair cortex, elongate, multiply and enter into the hair cortex as intrapilary hyphae. These hyphae cannot pass beyond the keratogenous zone and remain there in the form of a fringe called the Adomson's fringe [Figure 1]. Then, the hyphae start producing spores within the hair cortex (endothrix) [Figure 2] or on the surface of the hair cortex (ectothrix) [Figure 3], depending on the species involved in the infection. Majority of the anthropophilic spp. induce no inflammation, while the zoophilic species and occasionally few anthropophilic spp. can mediate an inflammatory reaction, producing boggy nodular swelling called kerion or crusted plaque-like lesions called favus. The inflammatory type involves only few hairs whereas more hairs are involved in the non-inflammatory type. Therefore, the infectivity is higher with the latter type. Histologically, the non-inf lammatory types show very little or no cell infiltrate in the dermis and the inflammatory types show polymorphous infiltrate due to the cell-mediated immune reaction through their antigens. The destruction of hair follicular wall and sparse hair structures along with foreign body and Langhan's type of giant cells may also be observed in the dermis in the inflammatory types. Ultimately, fibrosis may occur with some of the inflammatory types like favus and kerion if not identified and treated early. Women having tinea pedis due to *Trichophyton rubrum* are prone to develop a foreign body granuloma that rarely occurs in their legs, where the hair is repeatedly shaven. This condition is called Majocchi's granuloma, clinically evidenced by discrete follicular papulonodules on the legs.

Malassezia spp. are gaining more importance in trichology because of their pathogenic role in the etiology of pityriasis capitis simplex (Dandruff) through their immune-mediated reaction. They survive in sebum-rich areas such as scalp, face and upper trunk. Evidences support the fact that Malassezia acts upon the surface lipids to release oleic acid and other free fatty acids, which lead to not only a breach in the barrier function of the scalp but also a direct irritant effect on the scalp, contributing to the pathogenesis of scalp seborrheic dermatitis.[8] It is also hypothesized that Malassezia can induce keratinocytes to release pro-inflammatory cytokines such as interleukin-1 alfa and tumor necrosis factor-alfa. These agents also induce folliculitis (*Malassezia folliculitis*) due to the rupture of the follicular wall and the yeast escaping down from the infundibulum, giving rise to a foreign body granuloma in the dermis.

## TRICHOMYCOSIS DUE TO DERMATOPHYTES

Tinea capitis is the most common infection, which is usually seen in children in the age group of 5-10 years.[7] Male children are more commonly affected.[7] The infection usually occurs through the use of contaminated blades in the pilgrimage centers where ritual head shaves are carried out in large numbers, mostly in unhygienic and overcrowded surroundings. Infection also occurs through fomites like towels, combs, hair brushes and theater seats.[9]

**Figure 4:**
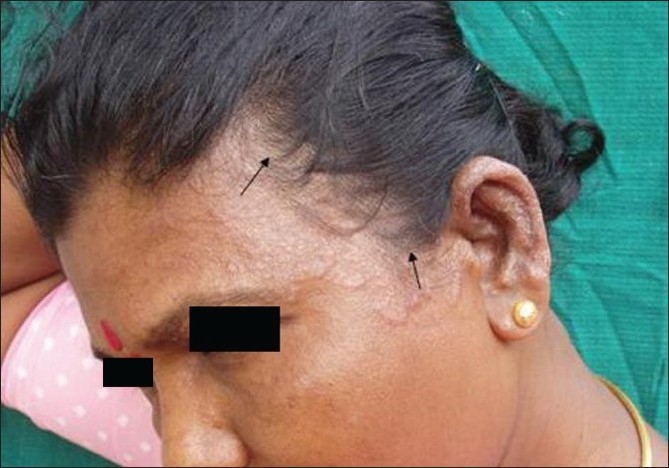
Glabrous type of tinea capitis, extension of infection from the forehead to the frontal region

**Figure 5:**
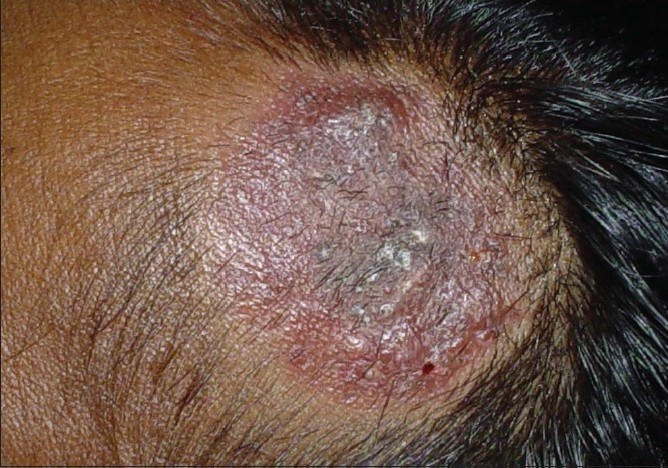
Kerion

The inflammatory types of tinea capitis include kerion, favus, abscess and pustular (agminate folliculitis) types. The non-inflammatory types are the grey patch, black dot, seborrhoeic, smooth patch of baldness and the glabrous type (also called the adult type) [Figure 4] of tinea capitis. In the latter type, extension of infection from the less-hairy skin to the scalp occurs, which is seen more in adults. Occasionally, one may see a mixture of all these types of tinea capitis in the same patient. Kerion is characterized by a boggy nodular swelling with follicular pustules [Figure 5] and, if left untreated, might give rise to scarring. The condition is painful and tender. Abscess type presents itself as a smooth erythematous boggy swelling without follicular pustules. The pustular type of tinea capitis is characterized by discrete or grouped follicular pustules. Favus is a special type of tinea capitis caused by *T. schoenleinii* and, less frequently, by *T. violaceum* and *M. gypseum* and manifests as initial yellow-red perifollicular papules with scaling and, eventually, with coalescence, producing a yellow cup-shaped concretion of mycelia along with cellular debris called scutula, with one or more long hairs seen projecting through the center. Scarring is always a sequel of favus infection.

**Figure 6:**
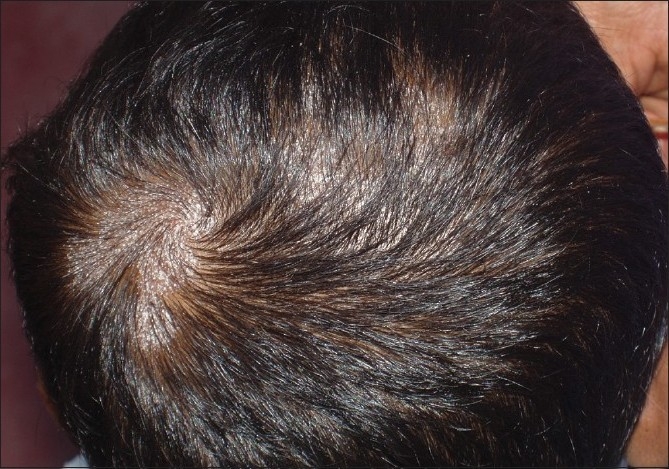
Grey patch type

**Figure 7:**
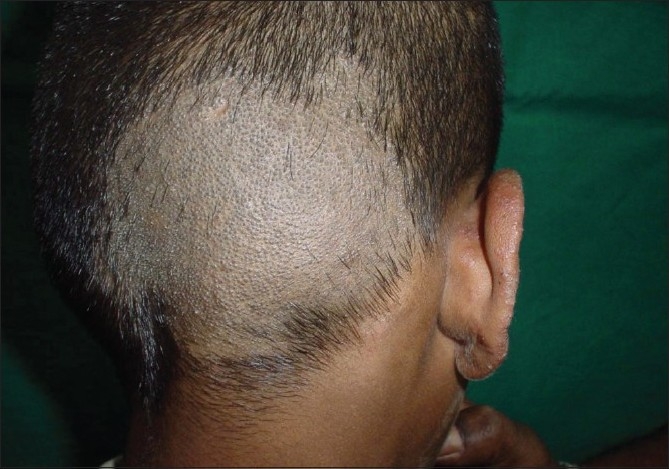
Black dot type with Tinea facei involving the pinna

The grey patch type of tinea capitis caused exclusively by anthropophilic agents is characterized by surface skin and hair infection with white scales along with broken bits of hair, a mixture of black and white resulting in the grey colour [Figure 6]. The black dot type of tinea capitis caused by *T. violaceum* and *T. tonsurans* shows minimal surface infection and hairs are predominantly involved and the broken stubs of hair on the scalp give the appearance of black dots [Figure 7]. Seborrhoeic type of tinea capitis is similar to seborrhoeic dermatitis in adults. Smooth patch of baldness resembling alopecia areata with minimal scaling also occurs in tinea capitis and hence all suspected cases of alopecia areata in children should be subjected for investigations to exclude tinea capitis.

In all types of tinea capitis, regional lymph nodes are enlarged in the cervical and occipital regions, which are painful and tender, especially in the inflammatory type of tinea capitis. Inflammatory tinea capitis is more often associated with 'id' reactions, which occur due to dissemination of fungal antigens in the blood stream tackled by the local immune mechanism in the skin. Although 'dermatophytids' can occur with any type of inflammatory dermatophytosis, it should be remembered to be observed in children with inflammatory type of tinea capitis, which might occur as papular eruptions on the trunk.

**Figure 8:**
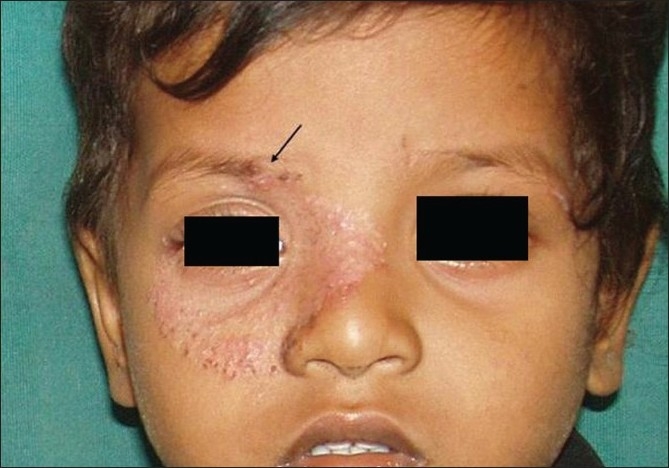
Tinea facei with involvement of the eyebrow and eyelashes

**Figure 9:**
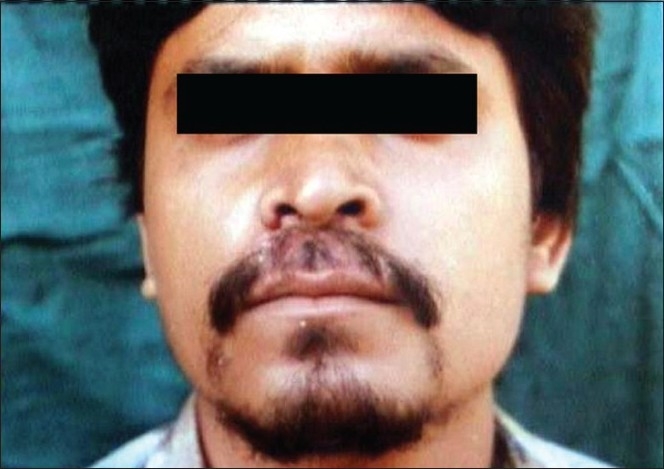
Tinea barbae with loss of hair

**Figure 10:**
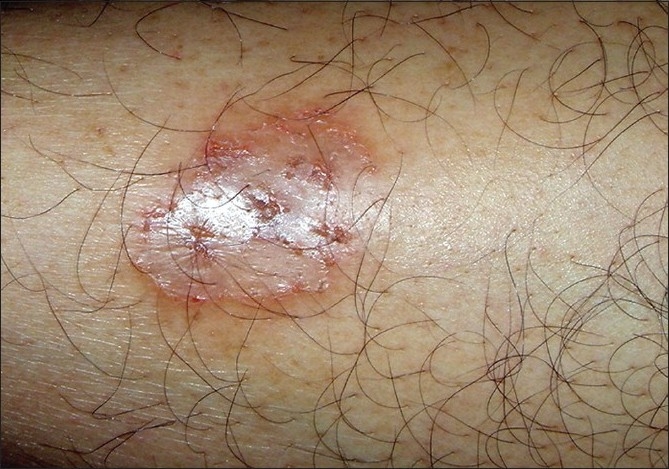
Tinea corporis with intra lesional papules suggestive of hair invasion

Tinea facei is also more frequently encountered in children, where the eyelashes and eyebrows may be involved [Figure 8]. Tinea barbae, the infection of beard region in adult men, also causes considerable concern because of the cosmetic disability of loss of hair [Figure 9]. Inflammatory lesions like kerion and favus can also occur in this region. Vellus hair infection should be looked for in the body sites other than scalp, which may appear as small papules within the lesion [Figure 10]. It is difficult to visualize hair infection on the body sites in inflammatory lesions, which should be confirmed only through wet mount preparation in potassium hydroxide (KOH) solution.

The varied presentation of trichomycosis due to dermatophytes depends on factors like the virulence and the type of the infecting organisms, the site of involvement, the type of hair infected, the immune status of the patient and the immune status of different sites in the same patient.

**Figure 11:**
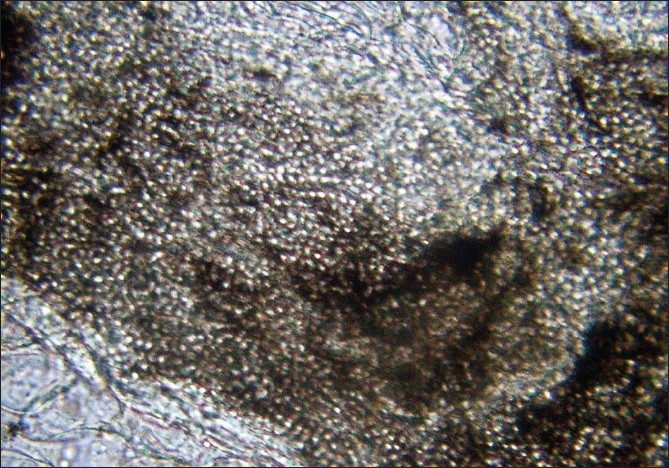
Endothrix spores in wet mount (KOH X400)

Trichomycosis due to dermatophytes could easily be diagnosed by an experienced dermatologist and investigations are necessary only for doubtful cases. Sampling of hairs in trichomycoses is important to confirm the diagnosis. Affected hairs easily come out from a kerion like a pin out of the cushion. In other types of tinea capitis, scraping the affected area with a blunt scalpel that yields affected hairs, broken-off hair stubs and scalp scales is preferable to plucking, which may yield uninvolved hairs. Alternatively, rubbing with a wet gauze or a sterile toothbrush also could yield the right specimen for culture.[10,11] The conventional 40% KOH wet mount under light microscopy, although not always positive, is confirmatory, which shows endothrix [Figure 11] or ectothrix spores, depending on the agents involved.

**Figure 12:**
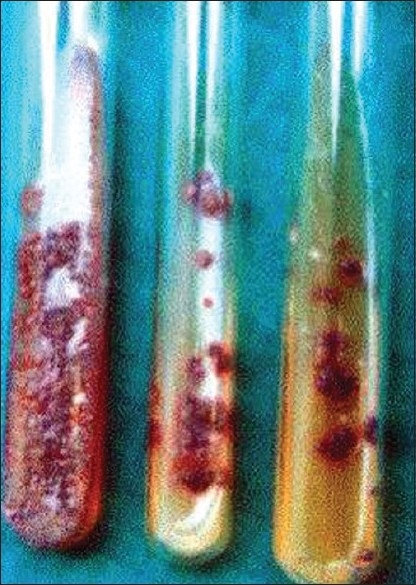
*Trichophyton violaceum* culture showing violet waxy colonies in culture

**Figure 13:**
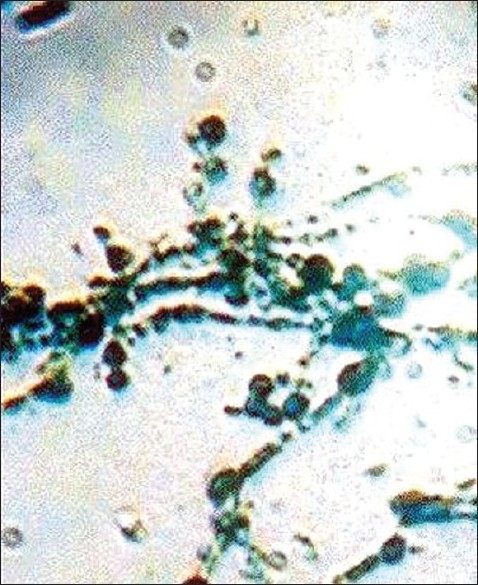
Microscopic colony morphology showing chlamydospores (LCB X1,000)

Cultures may be time consuming but offer accurate identification of the infecting agent, which may very often alter the course of treatment. Cultures are carried out in modified Sabouraud's dextrose agar medium to isolate the organisms. *T. violaceum* being the most common agent causing tinea capitis, produces violet waxy colonies [Figure 12] and the microscopic morphology shows plenty of chlamydospores [Figure 13].

**Figure 14:**
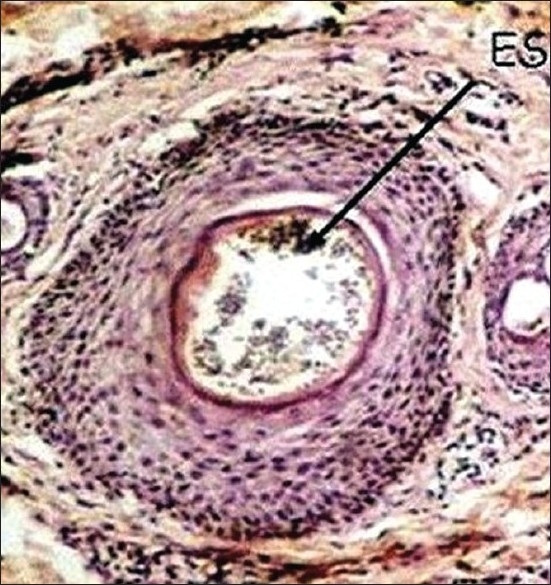
Endothrix spores within the hair cortex (H and E,× 400)

Polymerase chain reaction (PCR) using nested primers targeting the dermatophyte-specific sequence of chitin synthase 1 (CHS1) gene and modified PCR techniques have enabled rapid and specific diagnosis of trichomycosis.[12,13] Mycopathology and immunological investigations are rarely needed and are important only for academic purposes. Mycopathology of the infected hair reveals endothrix [Figure 14] or ectothrix spores in the infected hair. The inflammatory reaction in the dermis depends on the presence or absence of inflammation. Wood's lamp examination is not necessary in our part (Southern India) of the country, where the infecting agents are mostly non-fluorescent.

Management of dermatophytic trichomycosis is both topical and systemic. Topical application of antifungal lotions in the hairy sites of the body and shampoos like azoles, selenium sulfide, povidone iodine and zinc pyrithione (ZPT) in tinea capitis could reduce the spore load.[14] The topical agents that are useful in the hairy sites are the various azoles, ung. whitfield, tolnaftate, ciclopiroxolamine, terbinafine and buteneafine. Topical treatment alone is not sufficient and also not recommended.[15,16] But 2% ketoconazole shampoo as a monotherapy daily for 8 weeks has been reported to be successful in treating tinea capitis in children with clinical and mycological cure up to 1 year after treatment.[17]

Systemic antifungal therapy is often required to achieve a clinical and mycological cure rate as early as possible.[16] Griseofulvin in the dosage of 10-20 mg/kg body weight given at least for 8-10 weeks has remained as the licensed drug, economical and easily available, also as a syrup for children. Issues like prolonged treatment, long-term side effects, contraindications in pregnancy and liver disease, various drug interactions and the advent of newer relatively safer agents have limited the use of griseofulvin currently by many.

Terbinafine given in the dosage of 5 mg/kg body weight is effective against all dermatophytes. This could be considered as the systemic drug of choice for trichomycoses because this drug is fungicidal and therefore requires shorter duration of therapy with very minimal side-effects. Fluconazole 6 mg/kg and Itraconazole 6 mg/ kg could also be instituted, and the treatment duration is shorter with these latter drugs (4 weeks). Ketoconazole 4 mg/ kg for a period of 2-3 months may be used in adults but is not recommended in children because of the potential hepatotoxicity of the drug. Of these, griseofulvin alone is the licensed drug for the management of tinea capitis in children.[16]

## MALASSEZIA INFECTIONS

Malassezia spp. are lipophilic yeasts present as commensal microorganisms accounting for 40% of the scalp flora in normal individuals and found to play an important pathogenetic role in the pathogenesis of pityriasis capitis simplex, a mild form of seborrhoeic dermatitis of the scalp, accounting for 75-85% in seborrheic individuals. Malassezia derive their name from their discoverer, Malassez, who isolated them from dandruff scales in 1874 and implicated them as a causative agent of dandruff in 1889, renamed as *Pityrosporum malassez* by Sabouraud in 1904, again renamed as *P. ovale* and *P. orbiculare* based on culture morphology and in 1984 regained their original genus name Malassezia.[18]

Currently, 12 species of Malassezia have been identified,[19‐24] of which one (*M. pachydermatis*) is non-lipid dependent and all the others are lipid dependent [Table 2]. *M. globosa* seems to be the most common pathogen responsible for pityriasis capitis simplex, as shown by molecular techniques.[25] *M. furfur*, which was once thought to be the predominant causative agent based on culture techniques is now not present *in vivo*.

**Table 2:** Malassezia spp

*M. pachydermatis*
*M. furfur*
*M. globosa*
*M. restricta*
*M. obtuse*
*M. slooffiae*
*M. sympodialis*
*M. dermatis*
*M. equi*
*M. nana*
*M. yamatoensis*
*M. japonica*

*M. globosa* plays a pivotal role in damaging the scalp skin barrier, corneocytes hyperproliferation and scalp irritation through the release of oleic acid. The pro-inflammatory reaction triggered by this leads to subclinical scalp and hair follicle microinflammation,[26] which progresses to perifollicular fibrosis,[27] contributing to the etiopathogenesis of androgenetic alopecia. Dandruff scalps lose two to three times more hair than non-dandruff scalps, correlating with increased scalp hair shedding associated with carriage of *M. globosa*.[28] Scalp squamometry index shows a positive correlation between the percentage of hairs in the late telogen and the severity of dandruff.[29]

Malassezia folliculitis is relatively rare in the tropics and tends to occur more commonly in the temperate regions. Clinically, it resembles acneiform eruptions on the upper trunk and arms, which may occur along with the classical lesions of pityriasis versicolor.[30] The typical lesion is a molluscoid comedo papule with a central dell representing the follicle. Malassezia folliculitis tends to occur frequently in immunosuppressed individuals.[31] Eosinophilic folliculitis of human immunodeficiency virus and acquired immunodeficiency syndrome also shows marked colonization of Malassezia yeasts.[32]

Large numbers of Malassezia yeasts invade the dilated hair follicle, which is distended with keratinous material and is occluded.[33] Histopathological demonstration of spores in the dermis among the infiltrate is confirmatory, which are visualized by special stains such as Periodic acid Schiff's or Gomari's methane amine silver stains.

Treatment of pityriasis capitis simplex largely depends on antidandruff shampoos with active ingredients such as various azole antifungals, coal tar, sulfur, selenium sulfide or ZPT. Topical therapy apart from shampoos includes lotions, gels or mousses containing azoles, ciclopiroxolamine, terbinafine and butaneafine and, in resistant cases, systemic therapy is required for a longer duration. Systemic antifungal agents must be instituted with one of the various azoles like ketoconazole, fluconazole and itraconazole.

ZPT 1% in a shampoo base possessing cytostatic, antiproliferative and anti-inflammatory properties is believed to have direct inhibitory effects on *M. globosa*.[34] 1% ZPT was shown to have a comparable efficacy with 2% ketoconazole in dandruff.[35] Selenium sulfide is also reported to have anti-Malassezial effects[36] while coal tar and sulfur possess low anti-Mallasezial effects.[37]

Ketoconazole 2% shampoo with the maximum efficacy among azoles remains the mainstay of treatment in dandruff because it persists in the hair keratin up to 72 h after shampoo use and is safer in infants.[38] Combination of 2% ketoconazole with 1% ZPT has been shown to be very effective in pityriasis capitis simplex.[39,40] Ciclopirox olamine 1.5% has anti-Malassezial effects as effective as 2% ketoconazole.

## PIEDRA

Piedra (meaning stone in Spanish) occurs on the hair shaft as asymptomatic multiple black [Figure 14] and white nodules, with the hair breaking in bits at the level of the nodules. It was described in 1865 by Biegel[41] and was classified into two types, namely black piedra and white piedra by Horta[42] in 1911. Black piedra being the most common in tropical climates is caused by *Piedraia hortae*. White piedra being the most common in temperate and subtropical climates is caused by predominantly *Trichosporon beigelli*, which is now named as *Trichospron asahii*[42] and five other Trichosporon sp., listed in Table 3. Agents causing piedra involving hair shaft are in a state of saprophytic existence.

**Table 3:** Causative agents of piedra

Black piedra
* Piedraia hortae*
White piedra
* Trichosporon asahii*
* Trichosporon ovoides*
* Trichosporon inkin*
* Trichosporon mucoides*
* Trichosporon asteriodes*
* Trichosporon cutaneum*

Application of plant oils on wet hair is implicated as a favorable factor for piedra infection in the tropics, assisted by the high humidity. The source of infection is mostly the soil. Black piedra mostly affects scalp hair with darkly pigmented stony hard nodules firmly attached around the hair shaft, able to create a metallic sound on combing. White piedra affects any hair-bearing site, mostly beard and pubic hair, with soft creamy white gelatinous nodules loosely attached to the hair shaft. Both types of piedra invade the hair shaft and lead to hair breakage, posing considerable cosmetic morbidity.

**Figure 15:**
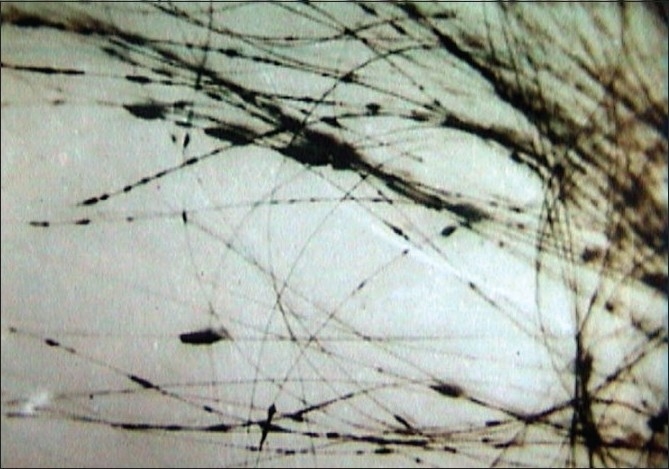
Black piedra-black nodule arranged irregularly on the hair shaft

**Figure 16:**
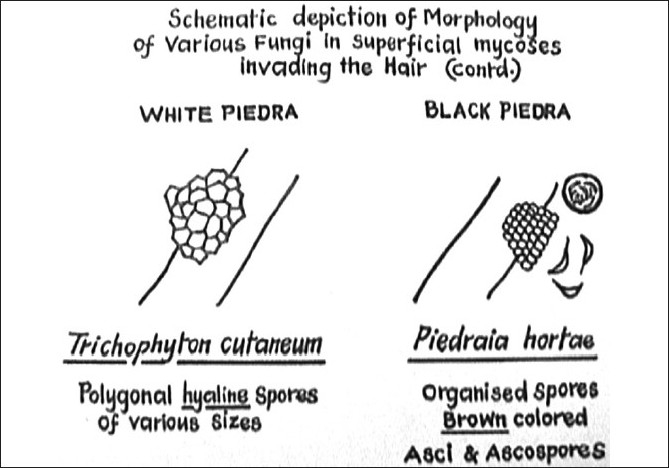
Schematic depiction showing the organized spores of black piedra, ascus, ascospores with flagella and irregular spores of white piedra

**Figure 17:**
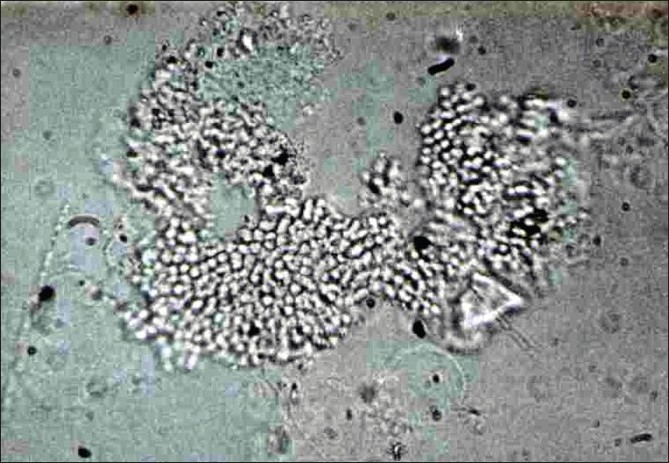
White hyaline spores of varying size of *Trichosporon* sp (white piedra)

The disease can easily be diagnosed clinically and wet mount of the nodules in KOH is confirmatory, which shows organized brown spores in black piedra invading the hair. Matured nodules will show asci (sexual spores) containing eight ascopores, each showing a small flagellum at their tip, which is better seen when the ascus ruptures. Grouped hyaline spores of varying sizes invading the hair are seen in white piedra [Figures 15‐17].

Treatment of piedra consists of avoidance of moisture, clipping of the affected hair, topical application of high concentration of terbinafine, tolnaftate, azoles and 1 in 2,000 mercury bichloride solution. Oral terbinafine can be used to treat black piedra. Oral itraconazole has been found to be useful in treating white piedra.[43]

## CONCLUSION

Dermatophytes and Malassezia spp. could be considered as common pathogens causing hair involvement compared to piedra, which is less frequent at least in our part of the country. With Malassezia, the involvement of the hair is secondary in the most commonly encountered pityriasis capitis simplex. Diagnosis of trichomycoses due to dermatophytes and piedra is simple, through wet mount of the skin scales and hair shaft in KOH. Pityriasis capitis is mostly a clinical diagnosis and Malassezia folliculitis needs histopathological confirmation. Response to therapy is total and complete in dermatophytic trichomycosis. Piedra takes a longer time to resolve while recurrence is most common with pityriasis capitis simplex, where other factors are also operative in addition to Malassezia spp., which colonize slowly following cessation of therapy.

## References

[1] Kamalam A, Nanjappa Chetty G, Balasubramanaian N, Chandrasekaran N, Thambiah AS (1979). Tinea capitis in a Moslem school. Indian J Med Res.

[2] Kamalam A, Thambiah AS (1979). Tinea capitis in South Indian families. Mykosen.

[3] Kamalam A, Thambiah AS (1980). Tinea capitis: An endemic disease in Madras. Mycopathologia.

[4] Kamalam A, Yesudian P, Thambiah AS (1977). Unusual presentation of *Trichophyton violaceum* Infection. Br J Dermatol.

[5] Chandler FW, Caplan W, Ajello L (1980). Superficial and cutaneous mycosis. 26. In: A colour Atlas and Text book of Histopathology of Mycotic Diseases.

[6] Orr GF (1969). Keratinophilic fungi isolated from soils by modified hair bait technique. Med Mycol.

[7] Kamalam A (1984). Observations on the Gamut/Galaxy of the fungal infections in TamilNadu state. Kavaka.

[8] Dawson T, Gemmer C, DeAngelis Y, Kaczvinsky J (2005). Dandruff and seborrheic dermatitis likely result from scalp barrier breach and irritation induced by *Malassezia* metabolites, particularly free fatty acids. J Am Acad Dermatol.

[9] Rippon JW (1988). Dermatophytosis and dermatomycosis. Cutaneous infections. Chapter 8. In: Medical mycology.

[10] Borchers SW (1985). Moistened gauze technique to aid diagnosis of tinea capitis. J Am Acad Dermatol.

[11] Fuller LC, Child FJ, Midgely G, Hay RJ, Higgins EM (1997). A practical method for mycological diagnosis of tinea capitis: Validation of the toothbrush technique. J Eur Acad Dermatol.

[12] Garg J, Tilak R, Garg A, Prakash P, Gulati AK, Nath G (2009). Rapid detection of dermatophytes from skin and hair. BMC Res Notes.

[13] Liu D, Coloe S, Baird R, Pederson J (2000). Application of PCR to the identification of dermatophytes fungi. J Med Microbiol.

[14] Neil G, Hanslo D (1990). Control of the carrier state in scalp dermatophytes. Pediatr Infect Dis J.

[15] Elewski BE (1996). Cutaneous mycoses in children. Br J Dermatol.

[16] Higgins EM, Fuller LC, Smith CH (2000). Guidelines for the management of tinea capitis. Br J Dermatol.

[17] Greer DL (2000). Successful treatment of tinea capitis with 2% ketoconazole shampoo. Int J Dermatol.

[18] Inamadar AC, Palit A (2003). The genus *Malassezia* and human disease. Indian J Dermatol Venereol Leprol.

[19] Gueho E, Meyer S (1989). A reevaluation of the genus *Malassezia* by means of genomic comparison. Antonie Leeuwenhock.

[20] Guilot J, Gueho E (1995). The diversity of *Malassezia* yeasts confirmed by rRNA sequence and nuclear DNA comparisons. Antonie Leeuwenhock.

[21] Gueho E, Midgley G, Guilot J (1996). The genus *Malassezia* with description of four new species. Antonie Leeuwenhock.

[22] Hirai A, Kano R, Makimura K, Duarte ER, Hamdan JS, Lachance MA (2004). A new yeast *Malassezia nana* sp.A novel lipid dependent yeast isolated from animals. Int J Syst Evol Microbiol.

[23] Sujita T, Tajima M, Takashima M, Amaya M, Saito M, Tsuboi R (2004). A new yeast *Malassezia yamatoensis* isolated from a patient with seborrheic dermatitis and its distribution in patients and healthy subjects. Microbiol Immunol.

[24] Sujita T, Takashima M, Shinoda T, Suto H, Unno T, Tsuboi R (2002). New yeast species *Malassezia dermatis* isolated from patients with atopic dermatitis. J Clin Microbiol.

[25] Gemmer CM, De Angelis YM, Theelen B, Dawson TL (2002). Fast non invasive method for molecular detection and differentiation of *Malassezia* yeast species on human skin and application of the method to dandruff microbiology. J Clin Microbiol.

[26] Mahe YF, Michelet JF, Biloni N, Jarrousse F, Buan B, Commo S (2000). Androgenetic alopecia and microinflammation. Int J Dermatol.

[27] Jaworsky C, Kligman AM, Murphy GF (1992). Characterization of inflammatory infiltrates in male pattern alopecia: Implication for pathogenesis. Br J Dermatol.

[28] Nematian J, Ravaghi M, Gholamrezanezhad A, Nematian E (2006). Increased hair shedding associated with the presence of *Malassezia*. Am J Clin Dermatol.

[29] Pierard-Franchimont C, Xhauflaire-Uhoda E, Loussouarn G, Saint Leger D, Pierard GE (2006). Dandruff associated smouldering alopecia: A chronobiological assessment of 5 years. Clin Exp Dermatol.

[30] Katoh T, Irimajiri J (1999). Pityriasis versicolor and Malassezia folliculitis. Nippon Ishinkin Gakkai Zasshi.

[31] Yohn JJ, Lucas J, Camisa C (1985). Malassezia folliculitis in immunocompromised patients. Cutis.

[32] Ferrandiz C, Ribera M, Barranco JC, Clobet B, Lorenzo JC (1992). Eosinophilic pustular folliculitis in patients with acquired immuno deficiency syndrome. Int J Dermatol.

[33] Back O, Faergemann J, Hornqvist R (1985). Pityrosporum folliculitis: Common disease of the young and middle aged. Am Acad Dermatol.

[34] Dawson TL, DeAngelis Y, Kaczvinsky J, Schwartz J (2004). Broadspectrum antifungal activity of pyrithione Zinc and its effect on the causes of dandruff and associated itch. J Am Acad Dermatol.

[35] Billhimer WL, Bryant PB, Murray KP, Coffindafer TW, Rains GY, Amon RB (1996). Results of a clinical trial comparing 1% pyrithione zinc and 2% ketoconazole shampoos. Cosmet Dermatol.

[36] Wattanakrai P, Arndt KA, Hsu JT (2007). Seborrheic dermatitis and dandruff. Manuel of dermatologic therapeutics.

[37] Gupta AK, Nikol K (2004). Use of sulfur in dermatology. J Drugs Dermatol.

[38] Brodell RT, Cooper KD, Wolverton SE (2001). Therapeutic shampoos. Comprehensive dermatological therapy.

[39] Saple DG, Ravichandran G, Desai A (2000). Evaluation of safety and efficacy of ketoconazole 2% and ZPT 1% in patients with moderate to severe dandruff: A post marketing study. J Indian Med Assoc.

[40] Patnaik RP, Desai A (2000). Clinical trial of ketoconazole 2% +ZPT 1% in dandruff. Indian Med Gazette.

[41] Beigel H (1869). The human hair. Its structure, growth, diseases and their treatment.

[42] Schwartz RA (2004). Superficial fungal infections. Lancet.

[43] Khandpur S, Reddy BS (2002). Itraconazole therapy for white piedra affecting scalp hair. J Am Acad Dermatol.

